# A combined physiological and biophysical approach to understand the ligand‐dependent efficiency of 3‐hydroxy‐4‐pyridinone Fe‐chelates

**DOI:** 10.1002/pld3.256

**Published:** 2020-08-16

**Authors:** Carla S. Santos, Andreia Leite, Sílvia Vinhas, Sofia Ferreira, Tânia Moniz, Marta W. Vasconcelos, Maria Rangel

**Affiliations:** ^1^ CBQF – Centro de Biotecnologia e Química Fina – Laboratório Associado Universidade Católica Portuguesa Escola Superior de Biotecnologia Porto Portugal; ^2^ REQUIMTE LAQV Departamento de Química e Bioquímica Faculdade de Ciências Universidade do Porto Porto Portugal; ^3^ REQUIMTE LAQV Instituto de Ciências Biomédicas de Abel Salazar Universidade do Porto Porto Portugal

## Abstract

Ligands of the 3‐hydroxy‐4‐pyridinone (3,4‐HPO) class were considered eligible to formulate new Fe fertilizers for Iron Deficiency Chlorosis (IDC). Soybean (*Glycine max* L.) plants grown in hydroponic conditions and supplemented with Fe‐chelate [Fe(mpp)_3_] were significantly greener, had increased biomass, and were able to translocate more iron from the roots to the shoots than those supplemented with an equal amount of the commercially available chelate [FeEDDHA]. To understand the influence of the structure of 3,4‐HPO ligand on the role of the Fe‐chelate to improve Fe‐uptake, we investigated and report here the effect of Fe‐chelates ([Fe(mpp)_3_], [Fe(dmpp)_3_], and [Fe(etpp)_3_]) in addressing IDC. Chlorosis development was assessed by measurement of morphological parameters, quantification of chlorophyll and Fe, and other micronutrient contents, as well as measurement of enzymatic activity (FCR) and gene expression (FRO2, IRT1, and Ferritin). All [Fe(3,4‐HPO)_3_] chelates were able to provide Fe to plants and prevent IDC but with a different efficiency depending on the ligand. We hypothesize that this may be related with the distinct physicochemical characteristics of ligands and complexes, namely, the diverse hydrophilic–lipophilic balance of the three chelates. To test the hypothesis, we performed an EPR biophysical study using liposomes prepared from a soybean (*Glycine3 max* L.) lipid extract and spin probes. The results showed that the most effective chelate [Fe(mpp)_3_] shows a preferential location close to the surface while the others prefer the hydrophobic region inside the bilayer.

**Significance statement:**

The 3‐hydroxy‐4‐pyridinone Fe‐chelates, [Fe(mpp)_3_], [Fe(dmpp)_3_], and [Fe(etpp)_3_], were all able to provide Fe to plants and prevent IDC. Efficacy is dependent on the structure of the ligand. From an EPR biophysical study using spin probes and liposomes, prepared from a soybean lipid extract, we hypothesize that this may be related with the distinct preferential location close to the surface or on the hydrophobic region of the lipid bilayer. [Fe(mpp)_3_] provide higher amounts of Fe in the leaves.

## Graphical abstract

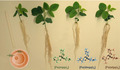

## 1 INTRODUCTION

Iron (Fe) is an essential nutrient for most living organisms including plants. Despite being the fourth most abundant element in Earth’s crust, Fe is only available in the environment in the form of very insoluble oxides and hydroxides, which are inappropriate for an adequate Fe uptake, in particular, in alkaline soils. Fe has a key role in fundamental biological processes, such as photosynthesis, chlorophyll synthesis, respiration, nitrogen fixation, enzyme activation, and electron transfer. When this micronutrient is unavailable to plants, they frequently develop yellowing of the younger leaves, exhibit reduced leaf areas and shoot and root dry weight (Santos et al., 2015). Iron Deficiency Chlorosis (IDC) is a major constraint for successful cultivation of crops in calcareous or alkaline soils around the world. Considering that *ca* 30 % of the world’s arable land lies in alkaline soils, farmers must rely on supplementing their crops with Fe to avoid severe growth deficiencies and disorders.

Plants can sense Fe deficiency and respond to the induced stress by triggering mechanisms in order to improve Fe uptake. A reduction‐based strategy (strategy I) and chelation‐based strategy (strategy II) have been identified as plants’ mechanisms for improving Fe uptake. Soybean, used as model in the present study, utilizes strategy I type mechanisms in which root Hþ‐ATPases acidify the rhizosphere so that Fe(III) solubility is increased, allowing Fe(III) reduction by membrane‐bound ferric reductases, like Ferric Reductase Oxidase 2 (FRO2). After reduction in Fe(III), Fe(II) is then absorbed into the root epidermal cells by Fe transporters, such as Iron‐Regulated Transporter 1 (IRT1; Morrissey and Guerinot, 2009).

Soybean is very susceptible to IDC and it has been used to study physiological and molecular mechanisms related to Fe uptake, transport, and accumulation (Roriz et al., 2014, Vasconcelos and Grusak, 2014). Various management strategies to correct Fe chlorosis are implemented in agriculture to increase yields (Wiersma, 2005, Liesch et al., 2011). The application of Fe fertilizers is effective in counteracting IDC of plants grown on calcareous soils and is the most commonly applied technique in agriculture (Lucena, 2006). The use of Fe salts is limited to low reactive media such as hydroponics or foliar applications due to their rapid precipitation under neutral‐alkaline pH, conditions that occur in calcareous soils. Traditionally, products based on synthetic Fe‐chelates, prepared from polyaminocarboxylate ligands such as EDTA (ethylenediamine tetraacetic acid) and EDDHA (ethylenediamine‐N, N0‐bis(o‐hydroxyphenylacetic), have been used to control and solve the problem of IDC (Rodríguez‐Lucena et al., 2010). However, although the use of polyaminocarboxylate synthetic Fe‐chelates in organic farming is legally permitted in the case of a severe deficiency of micronutrients, they do present some drawbacks, including environmental risks due to the persistence of the synthetic ligands in the environment (Nowack, 2002, Lucena, 2003) and only recently one biodegradable compound was reported (López‐Rayo et al., 2019). Therefore, an urgent need to test new Fe‐chelates with less impact on the environment and with properties that allow more efficient pathways for root uptake, root‐to‐shoot translocation, and maintenance of metal homeostasis is obvious.

In order to improve Fe uptake in Strategy I plants, an Fe‐chelate must be stable and it is known that its performance is largely determined by: (a) the ability of the ligand to maintain large amounts of Fe in solution and (b) the ability of the ligand to, once its original Fe has been delivered to the plant, take more Fe and supply it again to the developing tissues (López‐Rayo et al., 2009, Nadal, 2012).

Ligands of the 3‐hydroxy‐4‐pyridinone (3,4‐HPO) class are well known for their biological and analytical applications (Burgess and Rangel, 2008, Rangel et al., 2009, Moniz et al., 2011, Ferreira et al., 2018). The possibility of using the ligands in such a variety of fields is mainly due to their high affinity towards M(III) and M(II) metal ions and their versatility in synthesis, which allows preparation of chelators of variable denticity and distinct physicochemical properties (Leite et al., 2011, Moniz, Nunes et al. 2013, Moniz, Queirós et al., 2013). Since the molecules contain, in their chemical structure, both hydrophilic and hydrophobic parts, 3,4‐HPO ligands are considered amphiphilic molecules. For that reason, the concept of hydrophilic–lipophilic balance (HLB), originally defined for surfactants (Griffin, 1949), may also be applied to 3,4‐HPO ligands. Most ligands are non‐toxic and have been utilized in biomedical applications, namely, in the treatment of iron overloaded patients suffering from β‐thalassemia (Galanello, 2007). The structural features of the ligand, in particular, size and HLB, have proved to be of relevance for the efficiency of both the ligand and the complexes to achieve a particular biological effect (Galanello, 2007, Rangel et al., 2009, Moniz et al., 2011). The distinct interaction of structurally different ligands and complexes with biological membrane models has allowed rationalization of the dependence of the biological effect on the nature of the ligand (Galanello, 2007, Rangel et al., 2009, Moniz et al., 2011).

This family of ligands was considered as suitable to formulate new Fe fertilizers to address IDC in the sequence of our previous study in which a [Fe(3,4‐HPO)_3_] complex, [Fe(mpp)_3_], showed high efficacy on Fe chlorosis prevention in soybean (*Glycine max* L.) plants when compared to the commercial Fe chelate [FeEDDHA] (Santos, Carvalho et al., 2016).

**FIGURE 1:**
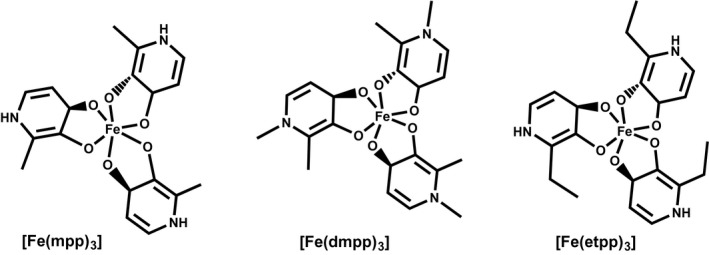
Formulae and abbreviations of Fe(III) chelates [(Fe(mpp)_3_], [Fe(dmpp)_3_], and [Fe(etpp)_3_]) in this work

To comprehend the ligand‐dependent efficiency of [Fe(3,4‐HPO)_3_] chelates in addressing IDC, we compared the effect of three structurally different complexes whose formulae and structure are shown in Figure 1 (([Fe(mpp)_3_], [Fe(dmpp)_3_], and [Fe(etpp)_3_]). Soybean (*Glycine max* L.) plants were grown hydroponically in controlled conditions and supplemented with the [Fe(3,4‐HPO)_3_] chelates. Upon a period of 14 days we analyzed several parameters in plants at a morphological, physiological, biochemical, and molecular level in order to compare their ability to deliver Fe to the plant.

Information about the interaction of biologically active molecules with biological membranes can be useful not only to understand their mechanism of action but also to infer about structure–activity relationships. Biophysical studies performed using liposomes as membrane models have been extensively used taking advantage of a set of spectroscopic techniques that provide information about the affinity of a molecule towards lipid bilayers and its preferential location within the hydrophilic and hydrophobic regions (Alves et al., 2016, Moniz et al., 2017, Rangel et al., 2018).

Electron Paramagnetic Resonance (EPR) spectroscopy is particularly valuable in this area of research since it allows the use of liposomes marked with spin probes located at the surface and deep inside the lipid bilayer. The ESR spectrum of each spin probe is sensitive to alterations in its molecular environment, thus, reporting the presence of molecules that are not part of the original bilayer. The analysis of the spectral features and EPR parameters of the probes in the absence and presence of the external molecule permits to get insight about its preferential location and permeation properties (Melnyk et al., 2016).

In the present work, the effect of the three Fe‐chelates ([Fe(mpp)_3_], [Fe(dmpp)_3_], and [Fe(etpp)_3_]) in addressing IDC was inspected by assessing the chlorosis development in hydroponically grown soybean plants. Also, an EPR biophysical study was performed using spin probes and liposome membrane models prepared from a soybean lipid extract, to get information on the distinct interaction of the [Fe(3,4‐HPO)_3_] chelates with membranes and understand the preferential location of the chelates in hydrophilic or lipophilic environments.

## 2 EXPERIMENTAL

### 2.1 Synthesis and characterization of Fe‐chelates

The 3,4‐HPO ligands Hmpp, Hdmpp, and Hetpp and their corresponding Fe(III) complexes, [Fe(mpp)_3_], [Fe(dmpp)_3_], and [Fe(etpp)_3_], were prepared in our laboratory following previously described procedures (Schlindwein et al., 2006, Queiros et al., 2011). Compounds were characterized by Elemental analyses (EA; C, H, N), ^1^H and ^13^C NMR spectroscopy, and UV‐vis spectroscopy. NMR spectra were recorded with a Bruker Avance III 400 spectrometer (400.15 MHz for ^1^H and 100.63 MHz for ^13^C) at Laboratório de Análise Estrutural, Centro de Materiais da Universidade do Porto (CEMUP; Portugal). Elemental analyses were performed at the analytical services of University of Santiago (Spain). The EA results for the Fe(III) chelates revealed that the complexes are obtained as hydrates and consistent with the formulae: [Fe(mpp)_3_]·4H_2_O, [Fe(dmpp)_3_].6H_2_O, and [Fe(etpp)_3_].6H_2_O Elemental analysis for C_18_H_18_N_3_O_6_Fe.4H_2_O, % calculated (% Found): C 43.22 (43.60) H 5.24 (5.23) N 8.40 (8.25); For C_121_H_24_N_3_O_6_Fe.6H_2_O, % calculated (% Found): C 43.61 (43.98) H 6.27 (6.31) N 7.27 (7.32); For C_18_H_18_N_3_O_6_Fe.1H_2_O, % calculated (% Found): C 51.66 (51.66) H 5.37 (5.54) N 8.61 (8.66).

### 2.2 Plant material, growth conditions, and treatments

Seeds of *G. max* cultivar “Williams 82” were germinated for 7 days in the dark at 25°C in moist paper. Germinated seedlings were transferred to 5 L vessels (five seedlings per vessel). The vessels were placed in a climate chamber (Aralab Fitoclima 10000EHF) with 16 h day photoperiod providing 325 µmol s^−1^ m^−2^ of photosynthetic photon flux density at plant level supplied by a mixture of incandescent bulbs and fluorescent lights. Temperature was set to 25°C during the light period and to 20°C during the dark period, whereas relative humidity was maintained at 75% throughout day and night. The standard solution for hydroponic growth of *G. max* included: 1.2 mM KNO_3_; 0.8 mM Ca(NO_3_)_2_; 0.3 mM MgSO_4_·7H_2_O; 0.2 mM NH_4_H_2_PO_4_; 25 mM CaCl_2_; 25 mM H_3_BO_3_; 0.5 mM MnSO_4_; 2 mM ZnSO_4_·H_2_O; 0.5 mM CuSO_4_·H_2_O; 0.5 mM MoO_3_; and 0.1 mM NiSO_4_. During the growing process, solutions were changed every 3 days.

Three different experiments were set up and all ended 14 days after transferring the plants to the climate chamber.

#### 2.2.1 Experiment 1 – Comparative evaluation of supplementation with [Fe(mpp)_3_], [Fe(dmpp)_3_], and [Fe(etpp)_3_]

Plants were grown in four vessels (n = 5) with the hydroponic solution described above and the addition of the different compounds, [Fe(mpp)_3_], [Fe(dmpp)_3_], and [Fe(etpp)_3_], at a final concentration of 20 µM, or no added Fe (‐Fe). Hydroponic solution was buffered with the addition of 1 mM MES [2‐(*N*‐morpholino)ethanesulfonic acid], at pH 5.5, as this is the optimum pH for nutrients absorption and to understand plants’ physiological and molecular responses (Li and Lan, 2015, Carrasco‐Gil et al., 2016, Ziegler et al., 2016).

#### 2.2.2 Experiment 2 – Examination of plant supplementation with [Fe(mpp)_3_] at lower concentrations

Plants were grown with Fe(mpp)_3_ supplementation in three vessels (n = 5) with three different concentrations: 20, 10, and 5 µM. Alike ‘Experiment 1’, hydroponic solution was buffered with the addition of 1 mM MES at pH 5.5.

#### 2.2.3 Experiment 3 – Examination of plant supplementation with [Fe(mpp)_3_] in alkaline conditions

Plants were grown with 20 µM [Fe(mpp)_3_] supplementation or with no added Fe (n = 5) with the hydroponic solution described above (two vessels) or buffered with the addition of bicarbonate buffer at pH 8.8 (two vessels).

### 2.3 Evaluation and analysis of the potential to prevent IDC

After 14 days of growth the plants were collected and the morphological and physiological parameters were measured. Samples for the several analyses were prepared according to the procedure detailed below for each parameter. Chlorosis development was assessed by measurement of morphological parameters, quantification of chlorophyll (SPAD) and Fe, and other micronutrients concentration (ICP‐OES). Measurements of enzymatic activity (FCR) and gene expression (FRO2, IRT1, and Leaf Ferritin) were also performed.

### 2.4 Morphological parameters

Sampled roots, stems, and leaves of the five biological replicates were separated, measured, and weighed.

### 2.5 Physiological parameters

Leaf chlorosis was assessed with Soil and Plant Analyzer Development (SPAD) readings, measured with a portable chlorophyll meter (Konica Minolta SPAD‐502Plus; Minolta, Osaka, Japan), using the youngest trifoliate leaf of five independent biological replicates.

### 2.6 Root iron reductase activity measurements

Root iron reductase was quantified as described before (Vasconcelos et al., 2006). The measurements were carried out in intact roots of five plants via the spectrophotometric determination of Fe^2+^ chelated to BPDS (bathophenanthroline disulfonic acid). Roots of each plant were submerged in assay solution containing: 1.5 mM KNO_3_, 1 mM Ca(NO_3_)2, 3.75 mM NH_4_H_2_PO_4_, 0.25 mM MgSO_4_, 25 mM CaCl_2_, 25 mM H_3_BO_3_, 2 mM MnSO_4_, 2 mM ZnSO_4_, 0.5 mM CuSO_4_, 0.5 mM H_2_MoO_4_, 0.1 mM NiSO_4_, 100 mM Fe(III)EDTA, and 300 mM BPDS. All solutions were buffered with 1 mM MES, pH 5.5. The assays were conducted under dim light conditions at 20°C and were terminated after 45 min by removal of the roots from the assay solution. Absorbance values were obtained at 535 nm, and an aliquot of the solution that had no roots during the assay was used as blank.

### 2.7 Determination of Fe contents and ionome study

The plant material was dried at 70°C until constant weight and 100 mg of dried plant tissue (root, stem, cotyledon, unifoliate, and trifoliate leaves) was mixed with 5 mL of 65% HNO_3_ in a Teflon reaction vessel and heated in a SpeedwaveTM MWS‐3+ (Berghof, Germany) microwave system. Each plant organ from all treatments (n = 5) was powdered and five independent digestions were carried out. The digestion procedure was conducted in five steps, consisting of different temperature and time sets: 130°C/10 min, 160°C/15 min, 170°C/12 min, 100°C/7 min, and 100°C/3 min. The resulting clear solutions of the digestion procedure were then brought to 50 mL with ultrapure water for further analysis. Determination of mineral nutrient concentration was performed using the inductively coupled plasma optical emission spectrometer (ICP‐OES) Optima 7000 DV (PerkinElmer, USA) with radial configuration.

### 2.8 Gene expression analysis

Plants grown for ‘Experiment 1’ were individually pulverized thoroughly with a mortar and pestle, until a fine powder was obtained, and total RNA was extracted using Qiagen RNeasy Mini Kit (#74904) according to the manufacturer’s instructions. RNA quality and quantity were checked by UV‐spectrophotometry, using a nanophotometer (Implen, Isaza, Portugal). Single‐stranded cDNA was then synthesized using First Strand cDNA Synthesis Kit (Fermentas UAB, #K1612) in a Thermal cycler (VWR, Doppio, Belgium), according to the manufacturer’s instructions. Sequence homologs to AtFRO2 and AtIRT1 in *G. max* were queried in NCBI database and the sequences with highest homology were selected. Primers for FRO2‐*like*, IRT1‐*like,* and ferritin were designed using Primer3 (Frodo.wi.mit.edu; Table S1, in electronic supplement). qPCR reactions were performed on a CFX96 Touch^TM^ Deep Well Real‐Time PCR Detection System (Bio‐Rad Laboratories Inc., CA, USA), using iQ^TM^ SYBR Green Supermix (Bio‐Rad Laboratories Inc., CA, USA) with the following reaction conditions: 95°C denaturation for 10 min; and 40 cycles with 15 s at 95°C, 30 s at 56°C–58°C (depending on primers used), followed by melt curve stages to check that only single products were amplified. The comparative CT method (∆∆CT; Livak and Schmittgen, 2001) was used for the relative quantification of gene expression values of Fe‐related genes using the geometric mean of the expression of the two stable reference genes (18S rRNA and actin) as controls transcripts and the plants grown with no added Fe as the reference sample. Two technical replicates were analyzed and data were transferred to Excel files and plotted as histograms of normalized fold expression of target genes.

### 2.9 Liposome preparation

A chloroform solution containing a lipid extract of soybean plant (Soy PC (20%)—Soy Total Lipid Extract—541601G Avanti), spin label (5‐DOXYL Stearic acid, ammonium salt—810612P Avanti and 16‐DOXYL‐stearic acid, free radical—253596 Sigma‐Aldrich), and the correspondent Fe‐chelate in a molar ratio of (lipid: spin label: chelate [10: 0.1: 1] or [10: 0.1: 0] for the control sample) were evaporated into dryness under a stream of nitrogen. The resulting film was left under vacuum for at least 3 hours to remove organic solvent traces. Multilamellar vesicles (MLVs) were obtained after re‐dispersion of the dried film with 20 mM PBS buffer (0.1 M NaCl, pH 7.4), and vortexed above the transition temperature. After this procedure, the MLVs were submitted five times to the following cycle: the vesicles were frozen in liquid nitrogen and the sample was thawed in a 37°C water bath. MLVs suspensions were then extruded 10 times on a Lipex Biomembranes extruder, trough polycarbonate filters (100 nm) to produce large unilamellar vesicles (LUVs).

### 2.10 Electron paramagnetic resonance spectroscopy

Electron paramagnetic resonance spectra were recorded at room temperature on a Bruker ELEXSYS E 500, equipped with an ER 4222SHQ resonator at Laboratório de Análise Estrutural, Centro de Materiais da Universidade do Porto (CEMUP; Portugal). The acquisition conditions used for the probe 5‐DSA were as follows: microwave power of 20 mW, magnetic field window of 150G (3285G to 3435G), modulation frequency of 100 kHz, modulation amplitude of 2G, gain of 60 dB, acquisition time of 50 ms, 45 scans, and magnetic field. The values of 2A_max_ (Neves et al., 2007) were determined from the experimental spectra.

The acquisition conditions used for the probe 16‐DSA were as follows: magnetic field window of 150G (3285G to 3435G) and microwave power of 20 mW, modulation frequency of 100 kHz, modulation amplitude of 2G, gain of 60 dB, acquisition time of 100 ms, and 2 scans. The values of the rotational correlation time were calculated from the experimental spectra.

### 2.11 Statistical analysis

Data were analyzed with GraphPad Prism version 6.00 for Windows (GraphPad Software, www.graphpad.com). For ‘Experiment 1’ and ‘Experiment 3’, differences between treatments were tested with ANOVA corrected for multiple comparisons using Holm‐Sidak method; for ‘Experiment 2’ differences were tested using Pearson correlation test. Statistical significance was considered at *p* < .05.

## 3 RESULTS AND DISCUSSION

The chemical structure of 3,4.HPO ligands is particularly attractive for biological applications since it allows tailoring of physicochemical properties of ligands and complexes without changing: (a) the affinity of a ligand towards a particular metal ion and (b) the redox potential of the metal ion complexes (Burgess and Rangel, 2008, Coimbra et al., 2019). The two latter properties are very important in what concerns the use of their Fe‐chelates as fertilizers, in particularly, for plants that use Strategy I for Fe uptake. First, for reduction strategy, the values of the redox potentials are crucial and, secondly, since coordination by 3,4‐HPOs is achieved through oxygen atoms, the chelators are hard ligands, thus, providing considerably higher stability constants for Fe(III) than for Fe(II; Burgess and Rangel, 2008). Distinct physicochemical properties, like size, polarity, and HLB of the complexes, may be important in what concerns their interaction with the radicular cell membrane.

In order to investigate the influence of the ligand on the effectiveness of the Fe‐chelates to prevent IDC, we chose the successfully tested compound [Fe(mpp)_3_] (Santos, Carvalho et al., 2016), and two others in which we varied: (a) the substituent on the nitrogen atom of the heterocyclic ring, [Fe(dmpp)_3_]), and (b) the substituent at position 2 of the heterocyclic ring, [Fe(etpp)_3_]. (Formulae and abbreviation of the Fe‐chelates are shown in Figure 1).

### 3.1 Studies in hydroponically grown soybean plants

#### 3.1.1 Experiment 1 – Comparative evaluation of supplementation with [Fe(mpp)_3_], [Fe(dmpp)_3_], and [Fe(etpp)_3_]

Soybean plants were grown in hydroponic controlled conditions as described in the experimental section and the three Fe‐chelates were used for Fe supplementation, and in agreement with previous work (Santos, Carvalho et al., 2016). After 14 days of growth, the plants were collected and the chlorosis development was analyzed through determination of key morphophysiological, enzymatic, and molecular parameters (Roriz et al., 2014, Santos et al., 2015).

**FIGURE 2:**
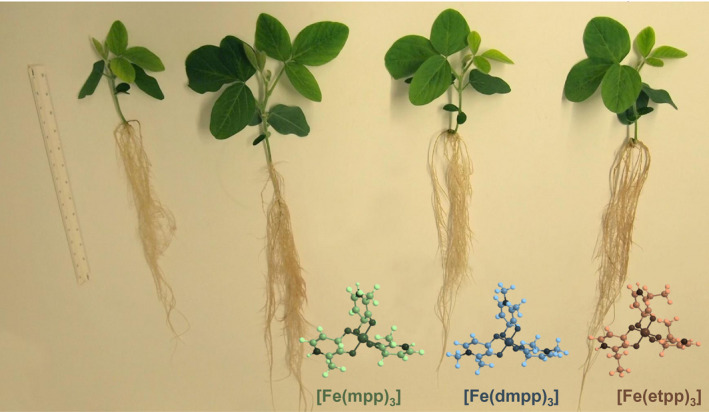
Effect of Fe(III)‐chelate treatments [no added Fe, Fe(mpp)_3_, Fe(dmpp)_3_, and Fe(etpp)_3_] on growth and morphology visual symptoms of *G. max* plants grown in hydroponic conditions

In Figure 2 a photo of the non‐treated and iron‐supplemented plants is shown to provide visual comparison of plants’ proportional growth and morphology.

**FIGURE 3:**
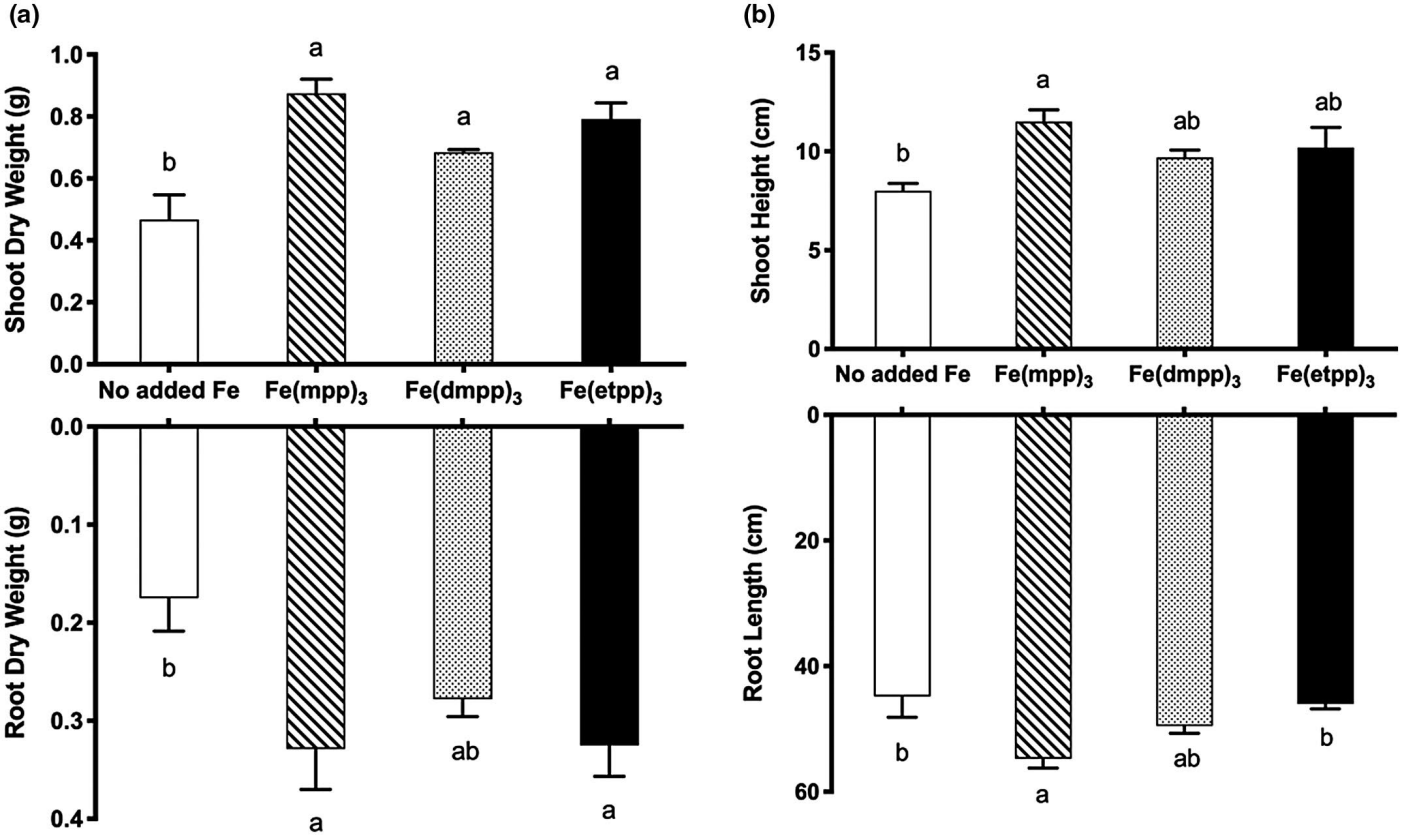
Effect of Fe(III)‐chelate treatments [no added Fe, Fe(mpp)_3_, Fe(dmpp)_3_, and Fe(etpp)_3_] on (a) dry weight (g per plant) of shoots and roots; and (b) shoot height and root length (cm) of *G. max* plants grown in hydroponic conditions. Data are means ± SE of five biological replicates. Significant differences between samples are indicated by different letters (*p* < .05) by ANOVA with Holm‐Sidak correction test

The quantification of the effect of Fe‐chelate treatments on dry weight of shoots and roots and shoot height and root length is displayed in Figure 3a,b, respectively. Plants’ growth was significantly improved for all Fe‐treated plants when compared to plants with no added Fe. In comparison with plants with no Fe supplementation, plants treated with [Fe(mpp)_3_] showed higher average dry weight (Figure 3a), with an increase of 47% in total biomass when compared to the no added Fe plants, as well as longer roots (Figure 3b). It has been reported that plants under nutritional stress usually increase their root biomass, which consists in larger area of secondary roots, in order to increase the nutrient absorption area, hence, resulting in larger but shorter roots [46,47]. This seems to be the case of treated plants with [Fe(etpp)_3_] that despite presenting root biomass similar to the plants treated with the other two compounds (Figure 3a) showed significantly shorter roots when compared to [Fe(mpp)_3_] (Figure 3b).

**FIGURE 4:**
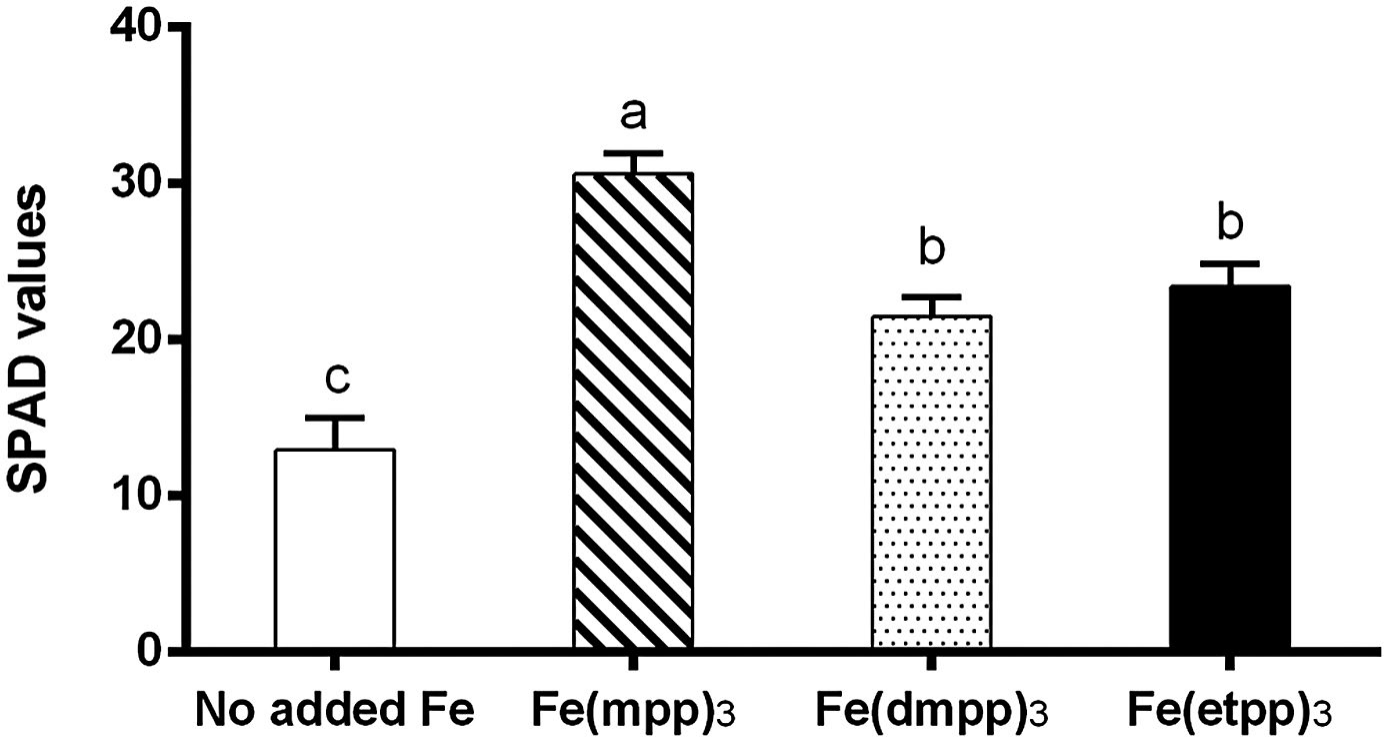
Effect of Fe(III)‐chelate treatments [no added Fe, Fe(mpp)_3_, Fe(dmpp)_3_, and Fe(etpp)_3_] on relative chlorophyll content (SPAD values) of *G. max* plants grown in hydroponic conditions. Data are means ± SE of five biological replicates. Significant differences between samples are indicated by different letters (*p* < .05) by ANOVA with Holm‐Sidak correction test

Due to the essential role of Fe in the chlorophyll biosynthesis (Santos, Serrão, et al., 2016), the yellowing of the leaves is one of the main visual symptoms of Fe deficiency. The relative chlorophyll content of the leaves was measured in SPAD values, which are shown in Figure 4. All Fe‐treated plants had higher chlorophyll accumulation than those that were not supplemented. In plants supplemented with [Fe(mpp)_3_], we observed a significant increase of 58 % (from 31 ± 1 to 13 ± 2 SPAD values), while in plants supplemented with [Fe(dmpp)_3_] and [Fe(etpp)_3_], this increase was ca 41 % (from 22 ± 1 to 13 ± 2 SPAD values) and 44 % (from 23 ± 2 to 13 ± 2 SPAD values), respectively. The results reinforce the previously observed efficacy of [Fe(mpp)_3_] (Santos, Carvalho et al., 2016), and seem to suggest that Fe delivered by this Fe‐chelate seems to be more bioavailable to the plants.

**FIGURE 5:**
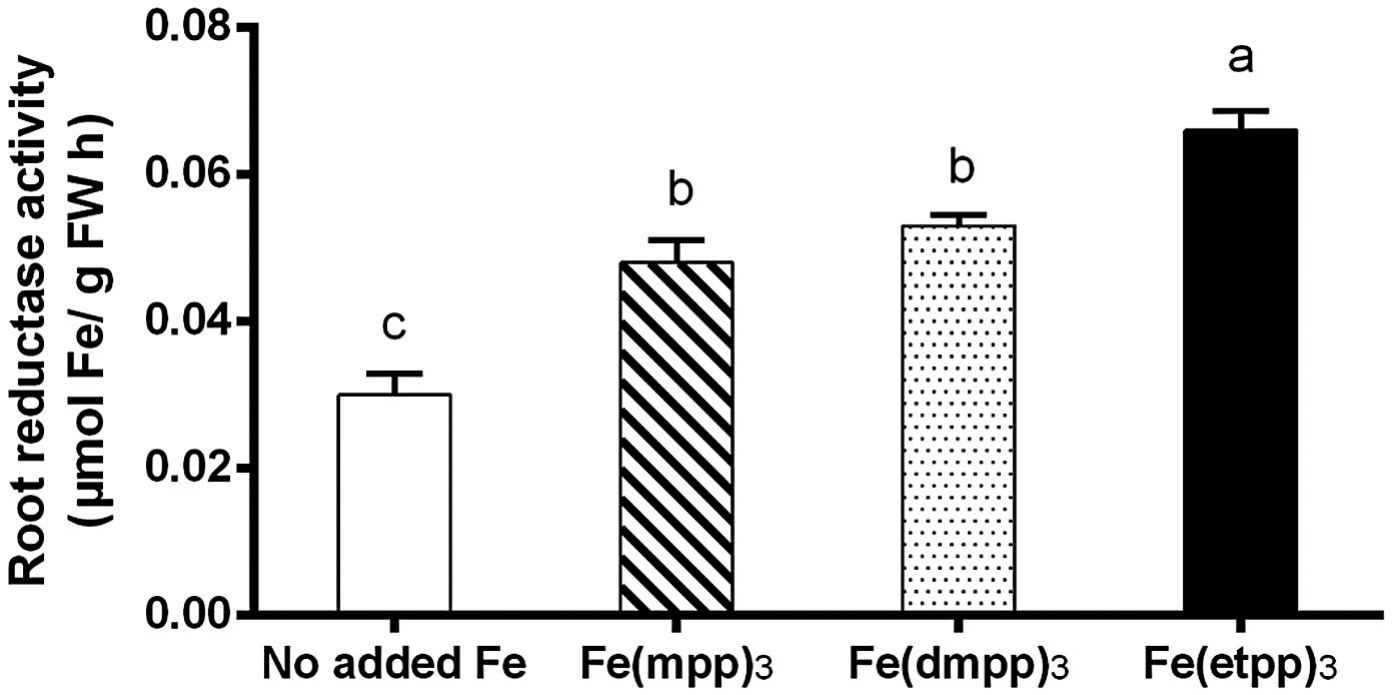
Effect of Fe(III)‐chelate treatments [no added Fe, Fe(mpp)_3_, Fe(dmpp)_3_, and Fe(etpp)_3_] on root reductase activity of *G. max* plants grown in hydroponic conditions. Data are means ± SE of five biological replicates. Significant differences between samples are indicated by different letters (*p* < .05) by ANOVA with Holm‐Sidak correction test

To confirm this observation, the enzymatic activity of root reductase enzyme, responsible for the reduction of Fe(III) to Fe(II) under stress conditions (Santos, Serrão, et al., 2016), was examined and the results are depicted in Figure 5.

We recognize that the lower value of root reductase activity obtained for plants grown without Fe supplementation can be misleading since plants in this condition are under severe stress and is not in agreement with studies is most plant species (Qiu et al., 2017). However, identical result has already been shown to happen in bean (Blair et al., 2010) and soybean (Santos et al., 2013), possibly due to the fact that the enzyme needs Fe for its functioning (Krishnan, 2005). For this reason, comparison of the activity of the enzyme is made between plant supplemented with the three Fe‐chelates. Roots of plants treated with [Fe(etpp)_3_] registered a significant increase when compared to those treated with [Fe(mpp)_3_] (17 %) and to [Fe(dmpp)_3_] (12 %) in root reductase activity, putatively demonstrating higher nutritional stress levels, a result which is coherent with the biomass results presented in Figure 3.

**FIGURE 6:**
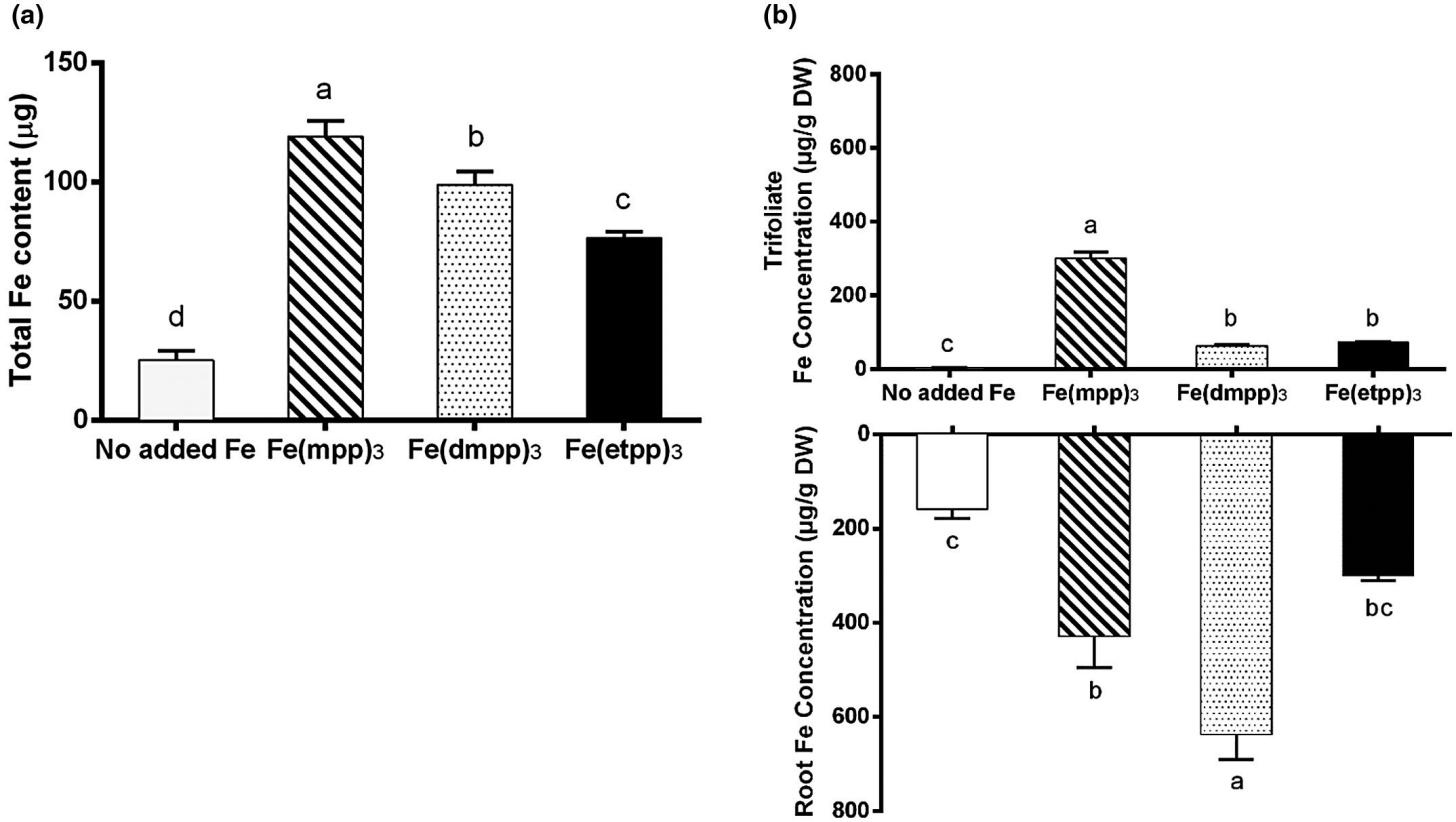
Effect of Fe(III)‐chelate treatments [no added Fe, Fe(mpp)_3_, Fe(dmpp)_3_, and Fe(etpp)_3_] on (a) total Fe content and (b) trifoliate and root Fe concentration of *G. max* plants grown in hydroponic conditions. Data are means ± SE of five biological replicates. Different letters indicate significant differences (*p* < .05) by ANOVA with Holm‐Sidak correction test

The bioavailability of Fe, in the form of the three distinct Fe‐chelates, was also analyzed by considering the Fe distribution profiles (Figure 6). The results of total Fe content (Figure 6a) show that supplementation with the Fe‐chelates efficiently provides Fe to the plants and also that plants supplemented with different Fe‐chelates exhibit a significantly different total Fe contents following the order [Fe(mpp)_3_]> [Fe(dmpp)_3_]> [Fe(etpp)_3_]. Compound [Fe(mpp)_3_] seems to be more efficient when compared to the other two Fe chelates. Specifically, compound [Fe(mpp)_3_] leads to an increase of 6 % in Fe content when compared to [Fe(dmpp)_3_] and 11 % when compared to [Fe(etpp)_3_].

The results show that the properties of the ligand are important for their efficacy in preventing IDC. The results obtained follow the same order of the [Fe(3,4‐HPO)_3_] chelates’ solubility in water [Fe(mpp)_3_]> [Fe(dmpp)_3_]> [Fe(etpp)_3_] (Burgess and Rangel, 2008). Alkyl‐3‐hydroxy‐4‐pyridinones show the expected decrease in water solubility as the sizes of the alkyl groups in positions 1 and 2 increase, thus, pointing out the influence of this property (Burgess and Rangel, 2008).

The values of the Fe content measured separately in roots and leaves are shown in Figure 6b and the results are very interesting. In plants supplemented with [Fe(mpp)_3_] the Fe content of leaves is significantly higher than that of the plants treated with the other compounds, for which the Fe content is similar. The result suggests that for plants treated with [Fe(mpp)_3_] Fe is translocated to the upper tissue in a considerable amount. This enhanced capacity of [Fe(mpp)_3_] to translocate Fe to the leaves was already observed when the effect of Fe‐chelate was compared with that of FeEDDHA (Santos, Carvalho et al., 2016).

Comparing the results obtained for Fe content in roots, for [Fe(dmpp)_3_] it seems that the most part of the detected Fe is accumulated in the root tissue (Figure 6b) while for [Fe(etpp)_3_] the result is much lower. The presence of the ethyl group in the latter compound seems to justify the lower Fe content as observed in Figure 6a.

In order to get information regarding the modulation of accumulation patterns of other mineral nutrients upon supplementation of plants with the Fe‐chelates, the concentration of several elements was measured in roots and shoots and is summarized in Table 1. Similarly to what was registered for Fe concentration (Figure 6b), when compared to plants with no added Fe, supplementation with [Fe(mpp)_3_] induced a significant accumulation of several other minerals in the leaves, namely, Zn, Mo, Mg, K, and Ca, while Fe(dmpp)_3_ led to the accumulation of Mg and K in the root tissue. The observed rise in the Mg concentration in the leaves of plants supplemented with [Fe(mpp)_3_] seems to be in agreement with the higher production of chlorophyll as indicated by the values of SPAD (Figure 4).

**TABLE 1:** The ionome of roots and trifoliate leaves of *G. max* plants grown with no added Fe or supplied with Fe(mpp)_3_, Fe(dmpp)_3_, and Fe(etpp)_3_ for 14 days under hydroponic conditions

Mineral (µg g^—1^)	Roots				Trifoliate leaves			
	No added Fe	Fe(mpp)_3_	Fe(dmpp)_3_	Fe(etpp)_3_	No added Fe	Fe(mpp)_3_	Fe(dmpp)_3_	Fe(etpp)_3_
Mn	31 ± 7^a^	53 ± 18^a^	77 ± 9^a^	63 ± 13^a^	79 ± 13^a^	64 ± 5^a^	40 ± 4^b^	57 ± 5^a^
Zn	210 ± 30^ab^	136 ± 19^b^	254 ± 9^a^	271 ± 37^a^	162 ± 22^b^	250 ± 14^a^	160 ± 14^b^	210 ± 17^a^
Mo	60 ± 18^a^	64 ± 18^a^	118 ± 5^a^	127 ± 22^a^	10 ± 4^b^	32 ± 3^a^	17 ± 3^b^	32 ± 1^a^
B	22 ± 4^a^	30 ± 2^a^	28 ± 1^a^	29 ± 1^a^	40 ± 13^a^	31 ± 1^a^	27 ± 1^a^	30 ± 1^a^
Na	2216 ± 62^a^	1112 ± 63^b^	1126 ± 61^b^	2340 ± 105^a^	1990 ± 236^a^	1450 ± 84^ab^	1421 ± 72^b^	1373 ± 50^b^
Mg	2224 ± 175^a^	2031 ± 540^a^	7576 ± 1421^b^	8947 ± 1610^b^	2670 ± 243^b^	5244 ± 764^a^	3007 ± 211^b^	3424 ± 262^b^
K	25.664 ± 6560^b^	39.072 ± 8053^ab^	59.386 ± 1420^a^	41.820 ± 12056^ab^	32.675 ± 4402^b^	60.662 ± 6896^a^	33.869 ± 3758^b^	41.142 ± 1804^b^
Ca	1695 ± 413^a^	2951 ± 844^a^	4569 ± 266^a^	3454 ± 1195^a^	12.722 ± 1238^b^	26.456 ± 3563^a^	14.873 ± 1017^b^	17.082 ± 711^b^
P	4720 ± 1143^a^	3583 ± 613^a^	5410 ± 213^a^	4715 ± 1350^a^	6318 ± 939^a^	6503 ± 762^a^	3522 ± 381^b^	5249 ± 76^ab^
Ni	12 ± 4^a^	18 ± 4^a^	25 ± 2^a^	18 ± 5^a^	10 ± 1^a^	15 ± 3^a^	8 ± 1^a^	11 ± 1^a^

Data are means ± SE of five biological replicates.

Different letters indicate significant differences (*p* < .05) within tissue types for each nutrient by ANOVA with Holm‐Sidak correction test.

**FIGURE 7:**
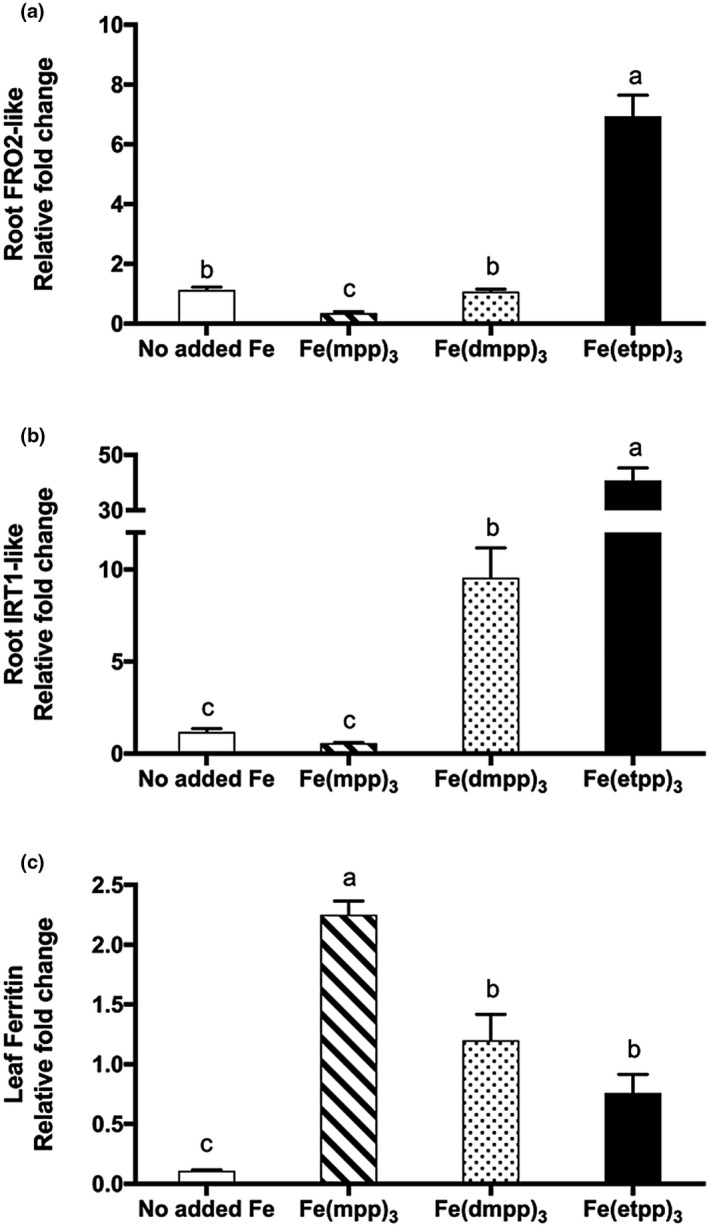
Effect of Fe(III)‐chelate treatments [no added Fe, Fe(mpp)_3_, Fe(dmpp)_3_, and Fe(etpp)_3_] on (a) root *FRO2*‐like; (b) root *IRT1*‐like; and (c) leaf *Ferritin* relative gene expression in *G. max* plants grown in hydroponic conditions. Data are means ± SE of five biological replicates. Different letters indicate significant differences (*p* < .05) by ANOVA with Holm‐Sidak correction test

After analysis of morphophysiological parameters, the relative expression of three genes (FRO2‐*like*, IRT1‐*like,* and ferritin) was evaluated, according to their importance in IDC response (Ivanov et al., 2012, Santos, Carvalho et al., 2016, Santos, Serrão, et al., 2016). FRO2 and IRT1 gene transcripts are usually accumulated in response to Fe stress conditions in order to increase Fe uptake (Jeong and Connolly, 2009, Xiong et al., 2014, Santos, Serrão, et al., 2016) and, as long as the plant keeps sensing this nutritional stress, the transcripts continue to be accumulated (Vert et al., 2003, Fuentes et al., 2018). Consistent with the general results obtained in this experiment, among the three Fe complexes, [Fe(mpp)_3_] induced the lowest transcript levels of FRO2‐*like* and IRT1‐*like* genes at the root level (Figure 7a,b). Also, as seen for reductase activity (presented in Figure 5), plants supplemented with [Fe(etpp)_3_] have the highest FRO2‐*like* and IRT1‐*like* gene expression levels (Figure 7a,b), putatively demonstrating increased stress levels. Again, plants grown with no added Fe presented low levels of these transcripts (Figure 7a,b) but, as mentioned before, Fe‐deficiency response gene expression is induced by Fe (Vert et al., 2003). The leaf expression levels of the *ferritin* gene (Figure 7c) followed the patterns registered for SPAD (Figure 4) and leaf Fe accumulation results (Figure 6b). Plants supplemented with [Fe(mpp)_3_] that had higher SPAD values and higher Fe accumulation in leaves exhibit the higher ferritin expression.

Overall, the results obtained in ‘Experiment 1’ shown that all three [Fe(3,4‐HPO)_3_] chelates are able to deliver Fe to plants, although their efficacy is different and dependent on the ligand. Chelate [Fe(mpp)_3_] is better in delivering Fe to plants, thus, supporting our hypothesis that although the ligands belong to the same class, the functional groups on their heterocyclic ring are important to select the Fe‐chelate with better capacity to enhance Fe uptake by plants and consequently prevent/treat IDC. Chelate [Fe(dmpp)_3_] is also promising to formulate a fertilizer and will be further studied.

#### 3.1.2 Experiment 2 – Examination of plant supplementation with [Fe(mpp)_3_] in different concentrations

Considering the efficacy of [Fe(mpp)_3_] in treating soybean plants for IDC, we conjectured if lower concentrations could also be utilized with similar results. The possibility of reducing the quantities of fertilizer necessary for IDC amendment would be very advantageous from the environmental and economical points of view.

In ‘Experiment 2’, we tested the impact of supplementation with [Fe(mpp)_3_] in three concentrations (20, 10, and 5 µM) on plants’ growth, relative chlorophyll content, and Fe accumulation profile (Table 2). No significant differences were found in total dry weight of plants treated with 20 and 10 µM concentrations, but differences were found for treatment with a 5 µM concentration of chelate. In what concerns SPAD results, for all concentrations, values were relatively high and no significant differences were found. Coherently with the SPAD results, no significant differences were registered between Fe chelate concentrations in leaf Fe accumulation but, as the concentration of Fe chelate was lower, a significant decrease in Fe accumulation in the roots was also observed (between 20 and 5 µM). It has been shown that supplementation with an Fe chelate at a moderate concentration might be more beneficial in Fe fertilization (Hasegawa et al., 2012), since it avoids formation of Fe oxides and consequently result in higher concentration of dissolved Fe (Bin et al., 2016). Given that, with 10 µM Fe chelate concentration, plants were able to produce a similar amount of biomass, to maintain similar levels of relative chlorophyll and to accumulate the same amount of Fe in leaves. In conclusion, it is possible to assert that chelate [Fe(mpp)_3_] may be an efficient Fe fertilizer, even at lower dosages (half a dose in this experiment) than those previously described for the compound (Santos, Carvalho et al., 2016), and for the commercially available fertilizers.

**TABLE 2:** Total dry weight, chlorophyll (SPAD values), total Fe content, and trifoliate and root Fe concentration ([Fe]) of *G. max* plants supplied with 20, 10, and 5 µM of Fe(mpp)_3_ for 14 days, under hydroponic conditions (‘Experiment 2’)

	[Fe(mpp)_3_] concentration		
	20 µM	10 µM	5 µM
Total dry weight (g)	1.8 ± 0.04^a^	1.5 ± 0.09^ab^	1.2 ± 0.14^b^
SPAD values	26 ± 0.6^a^	24 ± 0.5^a^	23 ± 0.7^a^
Total Fe content (µg)	120 ± 15^a^	99 ± 12^ab^	88 ± 10^b^
Trifoliate [Fe] (µg/g)	70 ± 6^a^	63 ± 4^a^	62 ± 6^a^
Root [Fe] (µg/g)	758 ± 26^a^	599 ± 26^ab^	487 ± 31^b^

Data are means ± SE of five biological replicates. Different letters indicate significant differences (*p* < 0.05) for each parameter by Pearson correlation test.

#### 3.1.3 Experiment 3 – Examination of plant supplementation with [Fe(mpp)3] in alkaline conditions

To be considered as an effective Fe chelate, [Fe(mpp)_3_] must be able to provide Fe to plants grown under neutral or alkaline conditions, which is the case of [FeEDDHA], commonly used in agricultural context (Lucena, 2006). In ‘Experiment 3’, plants were supplemented with [Fe(mpp)_3_] and grown hydroponically at both pH 5.5 and 8.8. Plants’ growth, relative chlorophyll content, root reductase activity, and Fe accumulation profiles were evaluated to understand the Fe chelate effectiveness at two pH values (Table 3). Comparison of the referred parameters for plants grown under pH 5.5 or 8.8 show that the total dry weight did not vary with the solutions’ pH, both in plants treated with no added Fe or with [Fe(mpp)_3_]. Chlorophyll content (SPAD values) was very low in plants with no added Fe in both pH conditions (Table 3). Plants supplemented with [Fe(mpp)_3_] were not affected by the pH variation.

**TABLE 3:** Total dry weight, chlorophyll (SPAD values), root reductase activity, total Fe content, and trifoliate and root Fe concentration ([Fe]) of *G. max* plants grown with no added Fe or supplied with 20 µM of Fe(mpp)_3_ at pH 5.5 or pH 8.8 for 14 days, under hydroponic conditions (‘Experiment 3’)

	No added Fe		[Fe(mpp)_3_]	
	pH 5.5	pH 8.8	pH 5.5	pH 8.8
Total dry weight (g)	0.75 ± 0.02^b^	0.71 ± 0.01^b^	1.99 ± 0.11^a^	1.85 ± 0.04^a^
SPAD values	7.23 ± 0.09^c^	12.24 ± 0.07^b^	27.44 ± 0.96^a^	26.56 ± 0.05^a^
Root reductase activity (µmol Fe/ g FW h)	0.02 ± 0.01^b^	0.04 ± 0.01^ab^	0.08 ± 0.05^a^	0.05 ± 0.03^ab^
Total Fe content (µg)	94 ± 14^b^	230 ± 16^a^	129 ± 12^b^	293 ± 30^a^
Trifoliate [Fe] (µg/g)	71 ± 3^c^	86 ± 3^bc^	100 ± 0.6^b^	133 ± 6^a^
Root [Fe] (µg/g)	263 ± 38^bc^	605 ± 33^a^	145 ± 6^c^	383 ± 19^b^

Data are means ± SE of five biological replicates. Different letters indicate significant differences (*p* < .05) for each parameter by ANOVA with Holm‐Sidak correction test.

As mentioned before, under alkaline conditions, root reductase activity is expected to increase, due to the hindering of the Fe reduction and uptake system (Blair et al., 2010), but in a recent study with sugar beet, the maximum reaction rate of a chloroplast ferric chelate reductase was in a pH range 6.5–7.0, decreasing to lower activity levels between 7.5 and 8.5 (Solti et al., 2014). Here, root reductase activity did not vary significantly with the pH, possibly because in plants grown at pH 8.8, the enzyme was not at its maximum reaction rate, maintaining about the same levels that those of plants grown at pH 5.5. Regarding the effect of pH on the Fe accumulation profile, total Fe content greatly increased in plants grown under alkaline conditions, accumulating mainly in the root tissue (Table 3). This Fe pool is most likely fixed in the root apoplast given the harsh pH conditions, as seen in maize in response to Fe deficiency (Shi et al., 2018).

The results demonstrated that the performance of chelate [Fe(mpp)_3_] at alkaline pH values is maintained, with no significant losses in biomass and chlorophyll levels, thus, preventing the development of IDC main symptoms.

### 3.2 Interaction of Fe‐chelates with model membranes

As previously stated, the chemical nature of the substituents on the heterocyclic ring of a 3,4‐HPO ligand (Figure 1) does not significantly influence its affinity for Fe or the redox potential of the corresponding Fe‐chelate, but it does modify properties like size and the HLB of both ligand and Fe‐chelate as demonstrated by studies regarding their solvation properties and *n*‐octanol‐water partitions coefficients (Burgess and Rangel, 2008, Santos et al., 2012). The HLB has been considered relevant to select 3,4‐HPO ligands for biomedical applications (Moniz et al., 2011, Moniz et al., 2017, Rangel et al., 2018) and interestingly it is also crucial for enhancement of Fe uptake in plants. In fact, the HLB has an important influence on the interaction of the molecules with biological membranes and can determine not only the uptake´ pathway but also the ability to cross‐membrane barriers within a cell (Moniz et al., 2017, Rangel et al., 2018).

To study the effect of different substituents on the interaction of several ligands and complexes with biological membranes, our group has been using a biophysical approach performed with liposomes as biological membrane models and spectroscopic methods (Fluorescence, NMR, and EPR; Moniz et al., 2017, Rangel et al., 2018). The information provided is, in our opinion, more realistic not only because it is possible to prepare liposomes with the appropriate type of lipids according to the target cell but also because the use of spectroscopic techniques permits to get detailed structural and topographical information concerning the preferential location of the molecule of interest.

**FIGURE 8:**
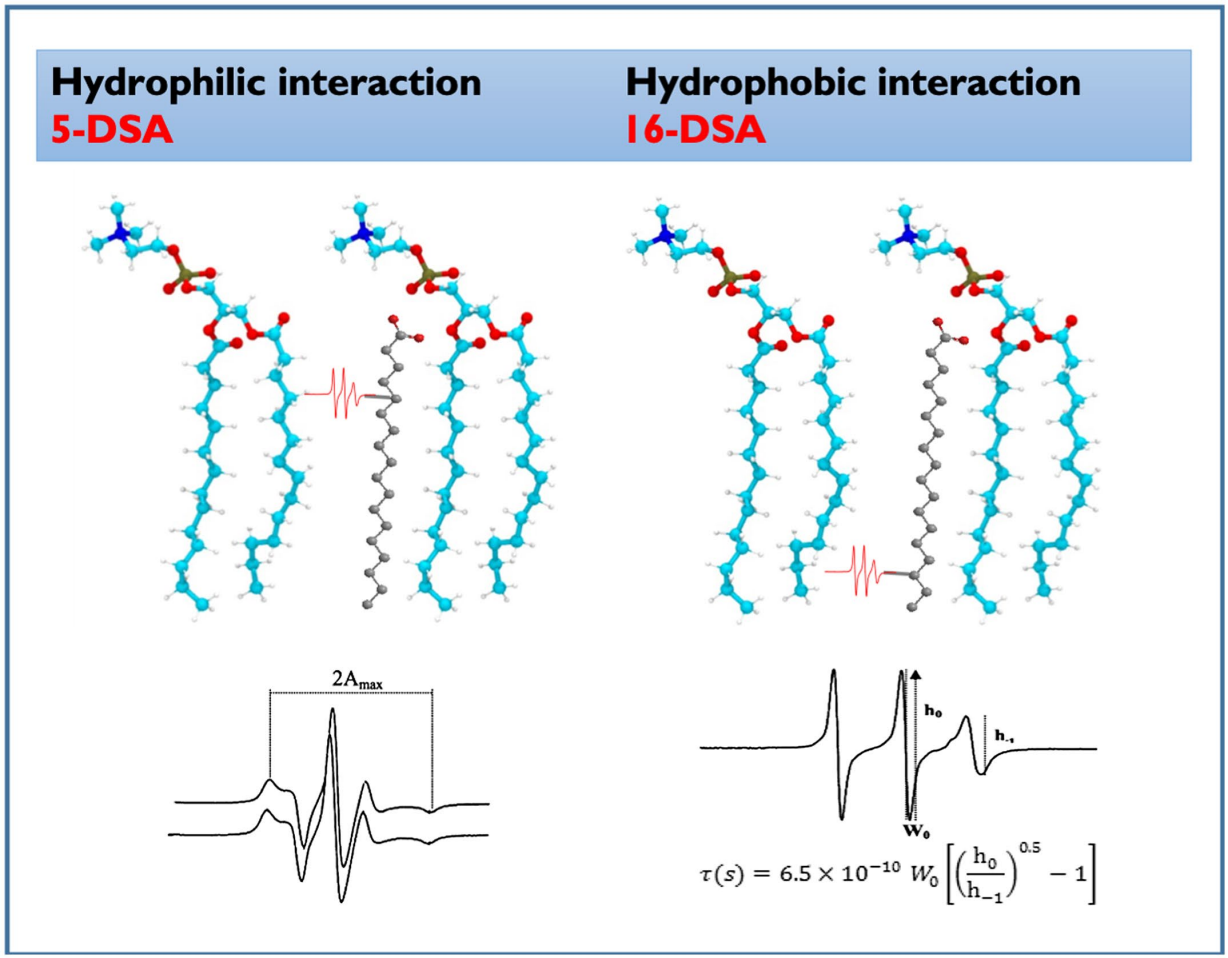
Spin probes at the water interface and inside bilayer of soybean extract liposomes. EPR parameters of the nitroxide radicals for 5‐DSA (degree of anisotropy (2A_max_)) and for 16‐DSA rotational correlation time τ

**FIGURE 9:**
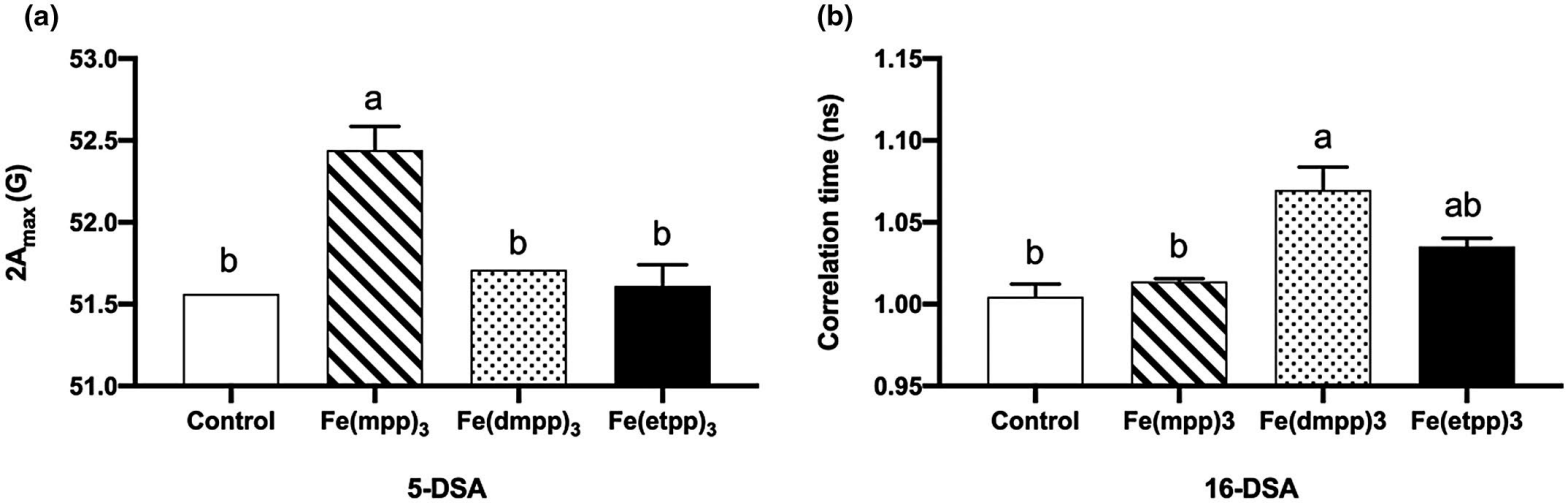
Effect of Fe(III)‐chelate treatments [control, Fe(mpp)_3_, Fe(dmpp)_3_, and Fe(etpp)_3_] on (a) 5‐DSA degree of anisotropy (2Amax) and (b) 16‐DSA rotational correlation time τ measured by EPR. Data are means ± SE of three replicates. Significant differences between samples are indicated by different letters (*p* < .05) by ANOVA with Tukey’s test

Considering the nature of the compounds and the application, we chose to prepare liposomes from a soybean lipid extract and incorporating nitroxide spin probes for EPR spectroscopic studies. As spin labels, we used the nitroxide probes, 5‐DSA and 16‐DSA (Figure 8), which report interactions at the surface and deep inside the bilayer, respectively. The EPR parameters of the nitroxide radical, degree of anisotropy (2A_max_) for 5‐DSA, and rotational correlation time τ for 16‐DSA are measured as shown in Figure 8 and the results obtained in the absence (control) and presence of the Fe‐chelates are shown in Figure 9.

As shown in Figure 9, chelate [Fe(mpp)_3_] strongly interacts with the probe 5‐DSA located in the polar region of the bilayer, a result which is in agreement with the presence of polar substituent at the nitrogen atom of the heterocyclic ring of the 3,4‐HPO ligand which favors hydrogen bonding. Chelate [Fe(dmpp)_3_] strongly interacts with the probe 16‐DSA located in the hydrophobic region of the bilayer, a result which can be accounted for considering the methyl group at the nitrogen atom of the heterocyclic ring of the 3,4‐HPO ligand that favors a preference for lipophilic regions. Chelate [Fe(etpp)_3_] although possessing a polar substituent at the nitrogen atom of the heterocyclic ring of the 3,4‐HPO ligand is much more lipophilic than [Fe(mpp)_3_] due to the presence of the ethyl group on position 2 of the heterocyclic ring. The interaction inside the bilayer is smaller than for [Fe(dmpp)_3_], although not statistically different. Overall the interaction of [Fe(etpp)_3_] with the liposomes is poorer than for the other two.

The results regarding the interaction of [Fe(3,4‐HPO)_3_] chelates with soybean liposomes have shown that the interaction and preferential location of the [Fe(3,4‐HPO)_3_] chelates with liposomes is dependent on the structure of the ligand. Compounds [Fe(mpp)_3_] and [Fe(dmpp)_3_] interact strongly with the lipid bilayer, although their preferential location seems to be different. Chelate [Fe(mpp)_3_] has higher affinity for the polar regions and locates mostly at the surface of the liposome, while [Fe(dmpp)_3_] has a higher affinity for the hydrophobic regions and locates frequently in the interior of the bilayer, a result which is consistent with its higher lipophilicity. The results for compound [Fe(etpp)_3_] suggested that its HLB does not favor a strong interaction with liposomes.

Considering the different preferential location of [Fe(mpp)_3_] and [Fe(dmpp)_3_], the results concerning their distinct total Fe content (Figure 7a) and Fe distribution in roots and in shoots (Figure 7b) we hypothesize that the two chelates may follow different pathways within the plant. We believe that in further experiments in which plants are grown for longer periods of time, namely, in the future experiments performed in soil, we will be able to investigate fluid composition and corroborate this preliminary hypothesis.

The higher affinity of the Fe‐chelate [Fe(mpp)3] for the surface of the membrane seems to favor it’s the rate of reduction and improve both uptake and translocation of Fe from the root to the leaves.

## 4 CONCLUSIONS

The results regarding the effectiveness of the [Fe(3,4‐HPO)_3_] chelates to prevent IDC demonstrate that: (a) all three chelates are able to deliver Fe to plants and (b) their efficacy is dependent on the ligand following the order [Fe(mpp)_3_]> [Fe(dmpp)_3_]> [Fe(etpp)_3_]. Chelate [Fe(mpp)_3_] provides the best effect as shown by the morphological, physiological, and gene expression parameters while [Fe(etpp)_3_] shows the poorer performance since even some stress signs are reckonable in plants treated with it.

The EPR results demonstrate that the interaction of Fe‐chelates with soybean liposomes is dependent on the ligand and is coherent with their IDC correction efficacy. The preferential location of the chelates [Fe(mpp)_3_] and [Fe(dmpp)_3_] for the surface and the interior of the bilayer, respectively, suggests that compounds may follow different pathways within the plants, thus, providing an explanation for the differences in Fe distribution. Also, preferential location at the surface may favor the uptake by the reduction strategy.

The studies performed for chelate [Fe(mpp)_3_] at variable concentration and pH values of the hydroponic culture medium demonstrate that the compound may be an efficient Fe fertilizer, even at lower dosages than those previously described (Santos, Carvalho et al., 2016) and used for the commercially available fertilizers. Chelate [Fe(mpp)_3_] showed a good efficacy in pH conditions simulating an alkaline soil even showing a better efficiency in providing Fe to plants as the increased Fe content values demonstrate.

The present investigation reinforces the potential of chelate [Fe(mpp)_3_] and puts forward chelate [Fe(dmpp)_3_] as new Fe fertilizers to prevent IDC. The knowledge gained from the distinct interaction of the two Fe‐chelates with biological membranes suggests new experiments to understand their pathways within plants and their mechanism of action.

## CONFLICTS OF INTEREST

None declared.

## AUTHOR CONTRIBUTIONS

Carla S. Santos designed the experiments, performed experimental work, analyzed results, and participated in manuscript writting. Andreia Leite, Sílvia Vinhas, Sofia Ferreira, and Tânia Moniz performed experimental work, analyzed results, and participated in manuscript writting. Marta W. Vasconcelos designed the experiments, analyzed results, and participated in manuscript writting. Maria Rangel conceived and designed the experiments, analyzed results, organized, wrote, and submitted the manuscript.

## Supporting information

Table S1
