# Supplementary material for: A combined physiological and biophysical approach to understand the ligand‐dependent efficiency of 3‐hydroxy‐4‐pyridinone Fe‐chelates
Source: Plant Direct. 2020 Aug 16;4(8):e00256. doi: 10.1002/pld3.256 (PMC7429444; doi:10.1002/pld3.256)
Supplement: Supplementary file 1 — Table S1 [file PLD3-4-e00256-s001.pdf]

A combined physiological and biophysical approach to understand the ligand-dependent efficiency of 3-hydroxy-4-pyridinone Fe-chelates

Carla S. Santos,[a] Andreia Leite[b], Sílvia Vinhas[b], Sofia Ferreira[c], Tânia Moniz[b], Marta W. Vasconcelos,[a] and Maria Rangel[c]*

[a] Universidade Católica Portuguesa, CBQF - Centro de Biotecnologia e Química Fina – Laboratório Associado, Escola Superior de Biotecnologia, Rua Diogo Botelho 1327, 4169-005 Porto, Portugal

[b] *REQUIMTE, LAQV*, Departamento de Química e Bioquímica, Faculdade de Ciências, Universidade do Porto, 4069-007 Porto, Portugal

[c] *REQUIMTE, LAQV*, Instituto de Ciências Biomédicas de Abel Salazar, Universidade do Porto, 4050-313 Porto, Portugal

*Corresponding author: mrangel@icbas.up.pt

Supplementary Information

Table S1. Primer sequences and correspondent accession numbers (Acc. No).

Primer	Forward (5'-3')	Reverse (5'-3')	Acc. No
18S	TTAGGCCATGGAGGTTTGAG	GAGTTGATGACACGCGCTTA	X75080.1
Actin	ATGCTCCTAGGGCTGTCTTT	GGGCTTCATCACCAACATAG	NC_016095.2
FRO2-like	CAGAACATGGAAGGGTCAAC	AGCAAGAACTCCCACACTTG	XM_003528793.2
IRT1-like	CTGAGGTTGTTCCTGGTGAG	TGCCAAGTCCTATCACCACT	KF542819.1
Ferritin	CAATGCTTCCTATGCGTACC	CTGAGGGGACATTCTTGATG	NP_001236534
